# The Hematological and Molecular Spectrum of α-Thalassemias in Turkey: The Hacettepe Experience

**DOI:** 10.4274/tjh.2014.0200

**Published:** 2015-05-08

**Authors:** Şule Ünal, Fatma Gümrük

**Affiliations:** 1 Hacettepe University Faculty of Medicine, Division of Pediatric Hematology, Ankara, Turkey

**Keywords:** molecular, mutation, α-Thalassemia, Turkey

## Abstract

**Objective:**

The spectrum of α-thalassemias correlates well with the number of affected α-globin genes. Additionally, combinations of the several non-deletional types of mutations with a large trans deletion comprising the 2 α-globin genes have an impact on the clinical severity. The objective of this study was to analyze the hematological and molecular data of 35 patients with Hb H disease from a single center in order to identify the genotypes of Hb H disease and genotype-phenotype correlations.

**Materials and Methods:**

Herein, we report the hematological and mutational spectrum of patients with Hb H disease (n=35). Additionally, genotypes of α-gene mutations of 78 individuals, who were referred to our institution for α-gene screening, were analyzed.

**Results:**

Supporting the previous data from Turkey, -α3.7 was the most common mutation among patients with Hb H disease (62.8%) and in the other 78 subjects (39.7%). Of the patients with Hb H disease, the most common genotypes were -α3.7/--20.5, -α3.7/--26.5, and -α3.7/--17.5 in 10 (28.6%), 6 (17.1%), and 6 (17.1%) patients, respectively. Another small deletion, -4.2 alpha, and several non-deletional types of α-gene mutations, namely α (-5nt): IVS-I donor site (GAG.GTG.AGG->GAG.G-----); α (PA-2): AATAAA>AATGGA, and α (cd59): GGC->GAC, were found to be associated with Hb H disease when present at trans loci of one of the large deletions given above. The combinations consisting of 1 non-deletional and 1 of the large deletional types of mutations (αTα/--) at trans loci were found to result in a more severe phenotype compared to the genotypes composed of 1 small trans deletion of a large deletion (-α/--). The combination of α (Cd59) and -- in trans was associated with severe phenotype and the disease was associated with an increase in Hb Bart’s level with null Hb H. In spite of the presence of 2 intact α-globin genes, homozygosity for PA-2 mutation resulted in severe Hb H disease.

**Conclusion:**

This study indicated that Hb H disease is not rare in Turkey and its genotype is quite heterogeneous.

## Abstract (tr)

**Amaç:**

Alfa (α) talasemilerin farklı klinik spektrumundan etkilenen α-globin gen sayısı sorumludur. Ayrıca delesyonel olmayan mutasyonların, iki α-globin geninin birden etkilendiği büyük delesyonel mutasyonlarla kombinasyon oluşturmasının da hastalığın klinik şiddetinde etkisi bulunmaktadır.

**Gereç ve Yöntemler:**

Burada Hb H hastalarımızın (n=35) hematolojik ve mutasyonel spektrumunu sunmaktayız. Buna ek olarak, merkezimize α-globin geninde mutasyon varlığı taraması için merkezimize gönderilen ve α-globin geni mutasyonu taşıyan 78 bireyin bulguları analiz edilmiştir.

**Bulgular:**

Çalışmamızda daha önce bildirilenleri destekler şekilde Hb H hastası grubunda (%62,8) ve 78 bireyde (%39,7) en sık mutasyon -α3,7 olarak bulunmuştur. Hemoglobin H hastalarımızda en sık genotipler -α3.7/--20.5; -α3,7/--26,5 ve -α3,7/--17,5 olarak sırasıyla 10 (%28,6), 6 (%17,1) ve 6 (%17,1) sıklıklarda bulunmuştur. Diğer bir küçük delesyon olan -4.2 (Asya tipi), delesyonel olmayan α-globin mutasyonları α (-5nt): IVS-I donor site (GAG.GTG.AGG->GAG.G-----); α (PA-2): AATAAA>AATGGA ve α (cd59): GGC->GAC; translarında büyük delesyonel bir mutasyon bulunduğunda Hb H hastalığına neden olduğu görülmüştür. Delesyonel olmayan mutasyonla büyük delesyonel tipte mutasyonların kombinasyonlarının (αTα/--), sadece delesyonel mutasyonların kombinasyonları sonucu gelişen Hb H hastalarına göre kliniklerinin daha şiddetli olduğu gözlenmiştir (-α/--). α(Cd59) ve -- trans birlikteliğinde, (α (Cd59)/--), daha ağır bir fenotip izlenmiştir ve bu durumda Hb H bulunmayıp, hastada Hb Bart’s yüksek olarak ölçülmüştür. Homozigot PA-2 mutasyonu olan hastalar (α PA-2/α PA-2) ağır fenotipte Hb H hastaları olarak gözlenmiştir.

**Sonuç:**

Çalışmamız Hb H hastalığının ülkemizde nadir olmadığına ve genotipinin heterojen olduğuna işaret etmektedir.

## INTRODUCTION

α-Thalassemia results from a genetic defect in α-globin chain synthesis, often as a consequence of deletional mutations and less frequently due to non-deletional types of mutations [1,2]. α-Thalassemias may occur worldwide; however, they are seen more commonly among populations in South East Asia, the Mediterranean region, and the Middle East [1]. The α-globin gene is located on the short arm of chromosome 16 (16p13.3) and normally there are 4 α-globin gene copies in an individual, with 2 in each allele [3]. The phenotype of α-thalassemias is directly related to the number of α-globin genes affected. α+-Thalassemias designate the status of deletion in one of the paired α-globin genes (-α/αα), whereas in α0-thalassemias both of the paired α-globin genes are deleted (--/αα). Heterozygous α+-thalassemia usually causes a silent carrier state. On the other hand, heterozygous α0-thalassemia (--/α) and homozygous α+-thalassemia (-α/-α) result in hematological findings similar to α-thalassemia trait, except for the Hb A2 value, which is at the normal level or below the normal level in α-thalassemia. The co-existence of both α+-thalassemia and α0-thalassemia (-α/--) results in hemoglobin H (Hb H) disease [1]. There are also non-deletional types of mutations (αTα) resulting in Hb H disease, when a large deletional type of mutation (--) co-exists in trans (αTα/--) [4,5]. 

The most common deletional mutations causing α+-thalassemia are -α3.7 and -α4.2, whereas the common deletional mutations causing α0-thalassemias are of 20.5-kb deletion, approximately 17.5-kb deletion (-MED-I), greater than 26.5-kb deletion (-MED-II), and approximately 18-kb deletion (-SEA) [1,4,6]. MED-II has previously been reported in a few Turkish families and from other Mediterranean populations [4].

In this study, the hematological and molecular data of 35 patients with Hb H disease from a single center were analyzed and reported in order to identify the genotypes of Hb H disease and genotype-phenotype correlations, and also to create awareness that Hb H disease is not a rare entity in Turkey.

## MATERIALS AND METHODS

Of the 788 patients who were diagnosed with thalassemia between 1981 and 2014 at our institution, 138 (17.5%) were diagnosed with Hb H disease (Table 1). Unfortunately, from those 138 patients only a total of 35 had genotype data available; those 35 were included in the current study. Splenomegaly was detected at diagnosis, during physical examination, or by ultrasonography in 40% of the patients with Hb H disease. The transfusion histories of patients with Hb H were recorded from patients’ files. Of the patients with Hb H disease, 18% received erythrocyte transfusion at least once, and 82% had no transfusion history at diagnosis and received no transfusion during follow-up. The number of transfusions ranged between 1 and 24. One patient was on a chronic transfusion program, whereas the other patients were transfused occasionally. Ethical committee approved this study.

Excluding the patients with Hb H disease, of the individuals screened for α-thalassemia mutations, 78 were found to carry an α-thalassemia mutation. The indications of α-thalassemia mutational screening among those 78 individuals were either having hypochromic microcytic erythrocytes, with normal iron status and Hb A2 below 3.5%, or being the available parent of a patient with Hb H disease. 

Results of hematological studies and red cell indices were analyzed. For discussion purposes, values prior to splenectomy or erythrocyte transfusion were taken into consideration. Hemoglobin A2, Hb F, and Hb H values were measured with the previously described methods [7] or high-performance liquid chromatography with the Bio-Rad Variant II system. Supravital stains for Hb H inclusions were examined in all cases [8]. 

Prior to 2008, α-thalassemia mutations were identified with previously described methods [7,8,9,10,11,12,13]. After 2008, mutation analyses for the α-globin gene were evaluated with the α-Globin Strip-Assay (ViennaLab, Austria), based on the reverse-hybridization technique used for detection of the 21 most common α-thalassemia mutations in the Mediterranean region. Of the 35 patients with Hb H disease, 25 have been reported previously [7].

The obtained data were evaluated with SPSS 21 (IBM Corp., Armonk, NY, USA). Normality test was performed to determine if the data were distributed in a normal fashion. For comparison between groups of more than 2, one-way ANOVA test was used. Statistical significance was determined as p values <0.05.

## RESULTS

Of the 35 patients with Hb H disease, the age range was 1.5-50 years at diagnosis (mean: 15.9±12.9 years). The mean values of red blood cell indices at diagnosis are summarized in Table 2a. A total of 10 different genotypes were detected in 35 patients with Hb H disease (Tables 2b and 2c.). 

Of the 35 patients with Hb H disease, 22 (62.8%) and 18 (51.4%) were found to have -α3.7 or --20.5 alleles, respectively (Table 3). The most common genotype was -α3.7/--20.5 in 10 (28.6%) of the patients, followed by -α3.7/--26.5 in 6 (17.1%) and -α3.7/--17.5 in 6 (17.1%). The most common 3 genotypes were distributed among 22 of the 35 patients, representing 62.8% of all genotypes found in patients with Hb H disease. The numbers of Hb H patients having other genotypes were too small to make any statistical analysis; therefore, comparison of the hematological data was made only among the patients with the 3 most common above-mentioned genotypes. 

Statistical analyses of the mean values of red cell indices showed no significant difference among these 3 common genotypes. Hemoglobin F level was found significantly higher in -α3.7/--17.5 patients (p=0.041), whereas Hb H levels were significantly lower among patients with this genotype compared to the -α3.7/--20.5 and -α3.7/--26.5 genotypes (p=0.036). Hemoglobin A2 levels were similar among these 3 genotypes. 

Of the patients with Hb H disease, 26 (74.3%) were found to have deletional types of mutations, whereas 9 (25.7%) were found to have non-deletional types of mutations. Comparison of the hematological data of the Hb H patients showed that the group of patients with a genotype consisting of non-deletional types of mutations with a large trans deletion (ααT/--) had statistically lower hemoglobin values (p=0.007) compared to those who had deletional types of mutations with a large trans deletion (-α/--) (Table 4). On the other hand, the mean of Hb H levels was significantly higher in the former patients (18.1± 8.3 vs. 7.4±4.7; p=0) than the latter (Table 4). In the examination of the 78 individuals with α-thalassemia mutations other than Hb H disease, the most common genotype was -α3.7/αα in 31 patients (39.7%) (Table 5). The most common non-deletional genotype was α(PA-1)/αα in 5 of the individuals (6.4%). Of the 78 subjects, 34 (43.5%) and 21 (26.9%) were found to have -α3.7 or --20.5 alleles, respectively (Table 5).

## DISCUSSION

The incidence of deletional α-thalassemia (-α/αα) among newborns screened by globin gene mapping from samples obtained from cord blood at birth has been reported to be 3.6% in Turkey [14]. In other reports, the chromatographic analyses of cord blood samples of newborns in Turkey suggested that -α/αα or (αTα) thalassemia incidence was between 2.9% and 4.1% [15,16].

In a recent report from Antakya-Hatay, a city in the southern part of Turkey, 300 individuals with moderate anemia, microcytosis, and normal iron levels were tested for α-thalassemia by the aid of α-globin strip assay; of these, 97 were found to have at least 1 mutation in 4 of the α-globin genes [17]. Of these patients, the most common mutation was -α3.7 (57.3%) [17]. Similarly, Öner et al. and Çürük reported -α3.7 as the most common α-thalassemia gene that was associated with Hb H disease in 25 and 32 patients, respectively [7,18]. Our study is compatible with the above stated previously published data pointing out that -α3.7 has been the most common genotype among patients with Hb H disease (62.8%). 

In our study, among Hb H patients, the second most common allele was --20.5 (51.4%). This finding is in accordance with the other reports from Turkey [7,18,19]. A hydrops fetalis case due to α-thalassemia associated with homozygosity of --20.5 was also previously reported from Turkey [20]. 

In the current study, the -MED-II deletion (--26.5) was found as the third most common allele among patients with Hb H disease (25.7%), which was followed by --MED-I deletion (--17.5) at 17%. Contrary to our observation, the --MED-I mutation (--17.5) has been reported as the second most common type of allele by Guvenc et al. with 15.11% frequency among the population of Adana, a city in the southern part of Turkey [21]. This is probably related to the homogeneity of the population studied in that publication. 

The --MED-II deletion has been known as a genotype more common among Turkish populations [4], and it was found as the third most common allele in our study.

All of these studies suggest that the molecular pathology of Hb H disease is heterogeneous and, according to our study, the most common genotypes associated with Hb H in 35 patients who were referred to us from all over Turkey are as follows: -α3.7/--20.5 (28.6%), -α3.7/--26.5 (17.1%), and -α3.7/--17.5 (17.1%) (Table 2b).

In the current study, 25.7% of the patients with Hb H disease who had a combination of large deletional and non-deletional (ααT/--) mutations were found to have statistically significantly lower Hb and higher Hb H levels compared to those of patients having combinations of large and small deletional (-α/--) types of mutations (Table 4). This finding was compatible with the previously published data [1,2,3]. This study revealed the presence of 3 different non-deletional types of mutations, namely the (-5nt), PA2, and C59 mutations. It seemed that the most common non-deletional type of combination involved in Hb H was (-5nt/--), which was found in 3 patients (8.6%) in the current study. Contrary to this, α (PA-2)/--MED-II was the most frequent non-deletional combination in a regional study by Çürük [18]. It was interesting that in spite of the presence of 2 intact α-globin genes, homozygosity for PA-2 mutation (α PA-2/α PA-2) resulted in severe Hb H disease in 2 patients (Table 2c); this was discussed elsewhere [7].

In this study, we did not find any of the previously described α-gene mutations from Turkey, such as -THAI, --FIL, init.cd, Cd 19, Hb Icaria, Hb Pakse, or Hb Koya Dora [14,16,17,18,19,21]. In a previous study from our center, the rate of unidentifiable mutations among individuals with α-thalassemia mutations was reported to be 2.72% [22]. In this study, all of the mutations among patients with Hb H disease were known mutations. In the previous study from our center, among individuals with α-thalassemia major, the most common 3 mutations were distributed among 69.39% of the patients [22]. In this study, it was shown that the most common 3 genotypes associated with Hb H accounted for almost 63% of the study group. 

In the previous reports by Altay and by Akar and Altay, related to National Hemoglobinopathy Registry data, Hb H was reported to be 3.6% (n=103) of all hemoglobinopathies in Turkey [22,23]. In our cohort study from a single center, it was shown that Hb H disease was diagnosed in 17.5% of the total 650 thalassemic patients (Table 1). The latest figure for α-thalassemia major in Turkey was reported to be 57% of 5500 patients with hemoglobinopathies [24,25]. Therefore, according to the data of our center as stated above, the total number of Hb H patients in Turkey should be around 550. The discrepancy in the rates of Hb H between 2002 data and the current study may derive from the higher awareness of the disorder in some centers in recent years, more accurate diagnoses, and/or developments in the diagnostic tools of Hb H disease and/or an increase in referral rates of anemic patients from peripheral to tertiary centers like ours. Therefore, if the figure of the current study reflects a more accurate value of the number of Hb H cases, we may expect to diagnose more patients in the near future.

In conclusion, as our center is a referral center in the mid-Anatolia region with a patient profile from all over the Turkey, the results of our study may represent the Hb H disease rates among the overall Turkish population. Some of the data of this study were in agreement with previous reports [7,8,9,16,17,18,19,20], and our current study also indicated that the molecular spectrum of α-thalassemias is quite heterogeneous in Turkey, as all together 9 deletional and non-deletional mutations and 10 combinations of them were found to be associated with Hb H disease. In previous reports, the mutational spectra were reported to be less heterogeneous among smaller populations, such as among Cypriots and Iraqi Turks [26,27]. Although in this study the molecular pathology of Hb H disease has been addressed, the frequencies of rare genotypes associated with α-thalassemia requires more patients and further population studies, since most of the individuals screened for that purpose in the current study were parents of the patients with Hb H disease, a limiting factor in prediction of the population frequencies of several genotypes. This study also showed that Hb H disease is not uncommon in Turkey; therefore, this disease should be kept in mind in discussion of microcytic anemias and all efforts should be made for correct diagnosis of α-thalassemias. Detection of new cases will be helpful in determining the allele frequencies of different α-thalassemia mutations.

## Figures and Tables

**Table 1:**
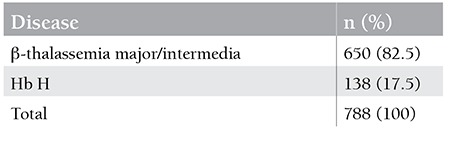
The distribution of β- and α-thalassemias between 1981 and 2014 in the Hacettepe University Division of Pediatric Hematology.

**Table 2a:**
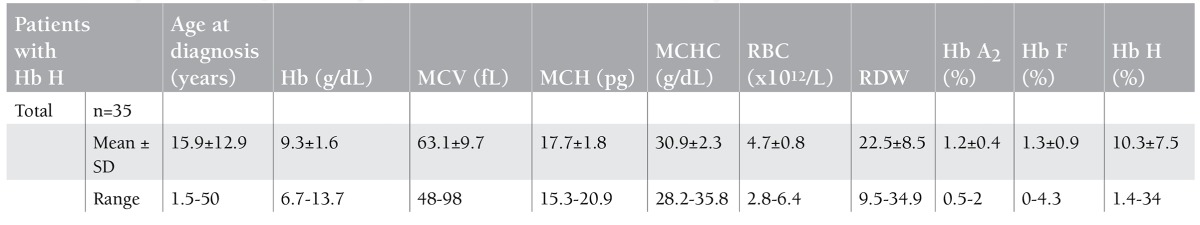
The age and hematological data of patients with Hb H disease with molecular diagnosis.

**Table 2b:**
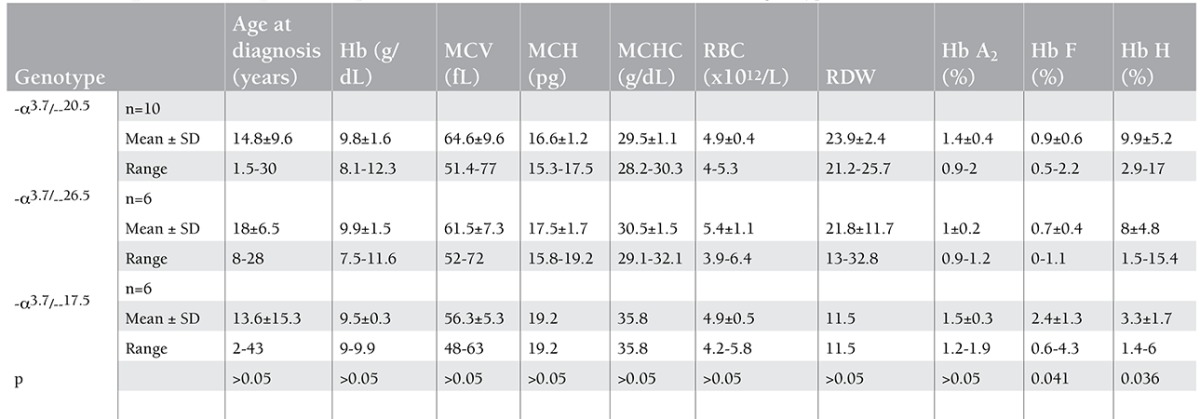
The age and hematological data of patients with Hb H disease with the 3 most common genotypes.

**Table 2c:**
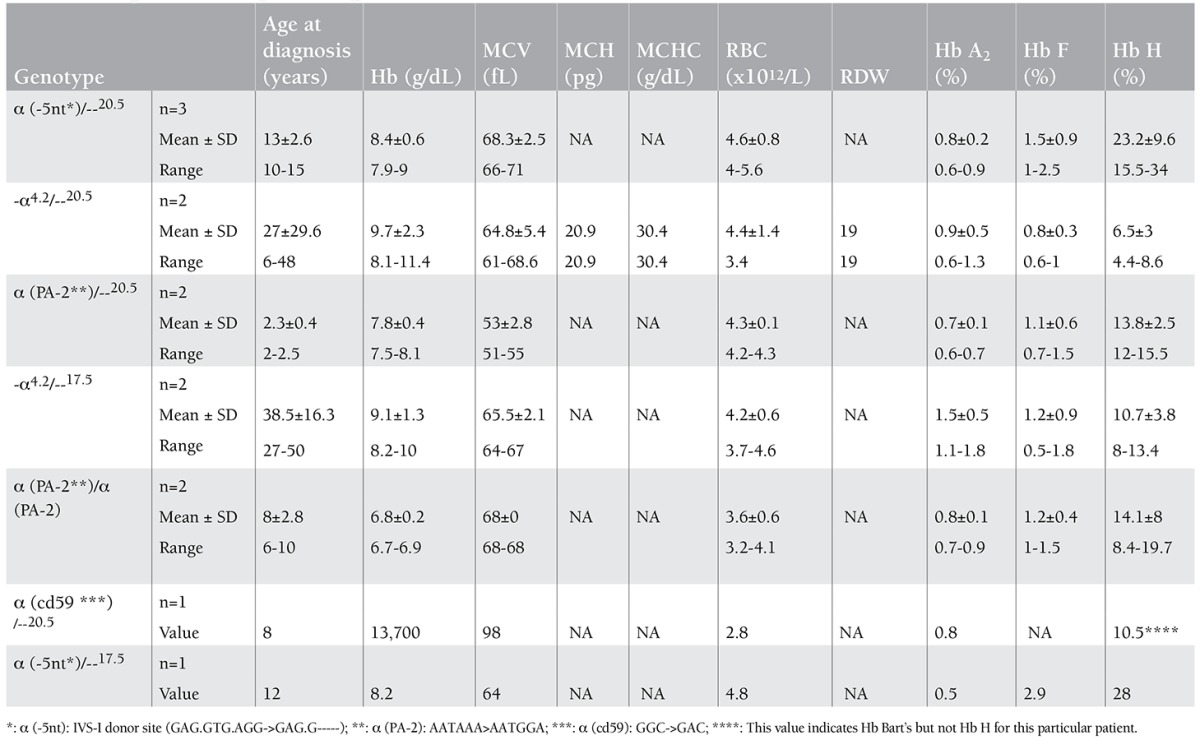
The age and hematological data of patients with Hb H disease associated with rare mutations.

**Table 3:**
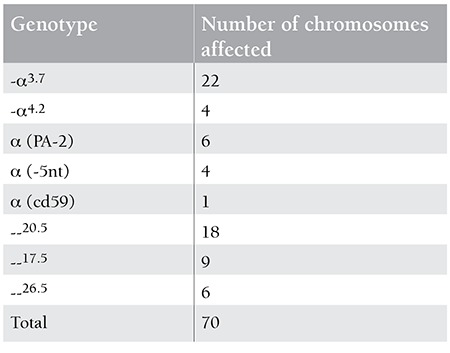
Distribution of mutations in 35 patients with Hb H (70 chromosomes).

**Table 4:**
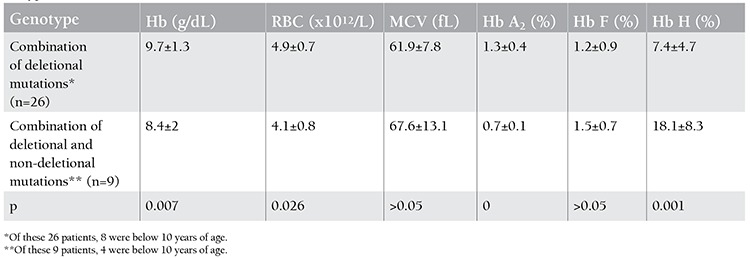
The comparison between hematological parameters of patients with Hb H disease with deletional and non-deletional types of mutations.

**Table 5:**
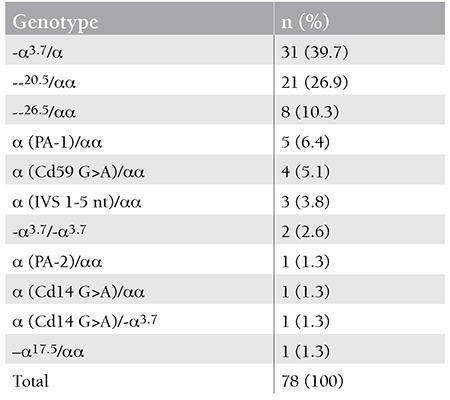
The distribution of deletional and non-deletional types of α-thalassemia mutations in 78 individuals.
